# Individually integrated traditional chinese medicine approach in the management of knee osteoarthritis: study protocol for a randomized controlled trial

**DOI:** 10.1186/1745-6215-12-160

**Published:** 2011-06-22

**Authors:** Cao Yuelong, Zhan Hongsheng, Pang Jian, Li Feiyue, Xu Shaojian, Gao Jinghua, Xu Zhanwang, Li Gang, Liu Ting, Guo Chaoqing, Shi Yinyu

**Affiliations:** 1 Research Institute of Orthopaedics, Shuguang Hospital Affiliated to Shanghai University of Traditional Chinese Medicine, 528 Zhangheng Road, Pudong New Area, 201203, Shanghai, China; 2 Department of Orhopaedics, Ruijin Hospital Affiliated to Shanghai Jiaotong University, 197 Rui Jin Er Road, Shanghai, 200025, China; 3 Department of Orhopaedics, GDHTCM Affiliated to Guangdong University of TCM, 111 Dade Road, Guangzhou, 510120, Guangdong, China; 4 Department of Orhopaedics, Wangjing Hospital Affiliated to China Academy of Chinese Medical Sciences, Huajiadi Street, Chaoyang District, 100102, Beijing, China; 5 Department of Orhopaedics, Hospital Affiliated to Shandong University of TCM, 42 West Culture Road, Jinan, 250011, Shandong, China

## Abstract

**Background:**

Knee osteoarthritis (OA) is considered a major public health issue causing chronic disability worldwide with the increasing number of aging people. In China and increasingly worldwide, many sufferers with knee OA are using complementary and alternative medicine including herbal drug, herbal patch, acupuncture and Tuina etc., to alleviate their symptoms. However, evidence gathered from systematic reviews or randomized controlled trials (RCT) has only validated acupuncture for the management of osteoarthritic pain. Moreover, such Traditional Chinese Medicine (TCM) methods above are commonly used in an integrative way. This trial is aimed to compare the efficacy of an individually integrated TCM approach in the management of knee OA with other single treatments as parallel randomized controls.

**Methods/design:**

Five teaching hospitals will participate in this randomized controlled trial. 500 participants, 100 in each hospital, will be randomly assigned to receive oral administration of a Chinese herbal drug (counter osteophytes capsule), topical use of a Chinese herbal patch (Fufnag Zijin patch), acupuncture, Tuina and the individually integrated TCM approach. The individually integrated TCM approach consists of basic treatment of oral counter osteophytes capsule, variable use of Tuina, acupuncture and a herbal patch based on the severity of the patient's symptoms.

The interventions are given for a period of 4 weeks. The primary outcome measure is the self-reported total score using the Western Ontario McMaster Universities Osteoarthritis Index (WOMAC). Secondary outcome measures include patient and investigator global assessment of response to treatment, patient and investigator global assessment of OA condition, WOMAC pain, stiffness, and physical function subscales, short-form 36 (SF-36) and TCM assessment of OA condition measured by syndromes questionnaire. Mixed models and sensitivity analysis will be used for the statistical analysis.

**Discussion:**

The trial is designed to test the hypothesis that an individually integrated TCM approach is more effective than four treatment modalities used separately. The major limitation of this study is lack of placebo control and of double blinding.

**Trial Registration:**

Chinese Cochrane Center ChiCTR-TRC-00000176

## Background

Osteoarthritis (OA), the most common form of arthritis, is a chronic, degenerative, joint disease that affects mostly middle-aged and older adults. OA is characterized by a series of pathological changes in the whole joint, including cartilage loss, bone remolding, excess synovial fluid secretion, capsular swelling, inflammation in the synovium (synovitis), bone marrow lesions, muscle weakness and atrophy resulting in bone thickening and formation of bone growths or spurs that interfere with joint movement [1,2].

Evidence gathered from a systematic review show that herbal medicines, which appear relatively safe, may offer a much-needed alternative, and merit further attention [3]. Current therapies in China available for the treatment of OA include: oral analgesics such as acetaminophen, ibuprofen, and topicals preparations including both Western medicine and Traditional Chinese Medicine (TCM) products. Acupuncture and Tuina have also been used for the management of OA. Although such TCM methods above have been regarded effective in clinical experiences and case reports, high level evidence has been rarely reported.

Only limited data are available on the efficacy and cost-effectiveness of Traditional Chinese herbs in the treatment of knee OA. According to TCM theory, OA is known as the Bi syndrome (painful obstruction), which means either the limbs or the joints are suffered from pain and malfunction; and herbal therapy has long been a standard treatment. The herbal drug can be used by external application or by oral administration. The counter osteophytes capsule, an extract from Radix Rehamanniae (Dihuang) and Herbal Cistanches (Roucongrong), is a Traditional Chinese herb used for symptomatic treatment of inflammation and pain in osteoarthritis. The earliest report was by Liu who reviewed the effect of counter osteophytes capsule on the treatment of OA in 1,000 cases [4]. Our previous study supported its efficacy in pain management of OA [5].

The Fufang Zijin patch, containing active ingredients extracted mainly from Kadsura Root-bark (Zijinpi) Negundo Chastetree Fruit(Huangjinzi) and Rhubarb (Dahuang), is also commonly used in China for the treatment of OA. According to TCM theory, the Fufang Zijin patch is indicated for the treatment of local joint pain, swelling, numbness, difficulty in physical activities due to the syndrome of "cold-dampness", "meridian obstruction", and "blood stasis".

Tuina is a very important component of TCM together with acupuncture and herbal medicines. Tuina, called 'An Mo' or medical massage in ancient times, usually refers to the doctor using their hands to manipulate the patient's body surface by means of various forms of tuina maneuvers such as pushing, kneading, rolling, wrenching, and flicking-plucking to relax and dredge the meridians and promote Qi flowing and blood circulating. Tuina is considered a natural treatment and manual therapy. It is distinct from chiropractic, osteopathic and mobilization because Tuina is used under the guidance of the basic theory of TCM. Among alternative care consumers with OA, the most commonly used treatment was massage therapy (57%) [6]. Some reports documente similar massage therapy as efficacious in the treatment of OA of the knee [7], but a systematic review of recent trials failed to confirm its efficacy [8].

In many Asian countries and increasing in western countries, acupuncture is a very popular treatment for OA. Systematic reviews and RCTs support the effectiveness of acupuncture for OA pain [9,10].

Optimal management of OA calls for an individual approach based on different constitutions of human body [11]. The individually integrated TCM approach in this study has been formed during 15 years of clinical experience in Shuguang hospital affiliated to Shanghai university of TCM. The approach consists of a basic treatment of orally administered Chinese herbal drugs and varying use of Tuina, acupuncture and herbal patch, all based on the severity of the patient symptoms.

We therefore initiated this study to compare the efficacy of an individually integrated TCM approach, Chinese herb counter osteophytes capsule, Fufang Zijin patch, acupuncture and Tuina in the short-term, symptomatic treatment of OA of the knee. We hypothesized that the integrated TCM approach is more effective than the four treatments, oral medication, topical patch, acupuncture or Tuina, when used alone.

## Methods/design

### Study design

This is a prospective, five-center, parallel-group, randomized controlled efficacy and safety study of individually integrated TCM approach in subjects with knee OA. After giving informed written consent, patients undergo a treatment period of four weeks. Ethics approval was received from Chinese Cochrane Center with the NO:ChiCTRCTET2008-9.

### Participants

All outpatients between 38 and 75 years of age who suffer from painful osteoarthritis of the knee according to American College of Rheumatology (ACR) criteria [12] will be screened. Additional inclusion and exclusion criteria are detailed in Table 1.

**Table 1:** Additional inclusion and exclusion criteria

Additional inclusion criteria	
Pain intensity of 20 mm or more by VAS in the previous 48 hours when walking on a flat surface; At least grade II to III on the Kellgren-Lawrence scale by an X-ray taken within 6 months;	And reluctant to continue on previous drug but willing to change drug treatment.

Exclusion criteria	
Patients presenting with any of the following will be excluded from the study.	

Grade I or Grade IV severity of the index knee based on the Kellgren and Lawrence radiographic criteria as applicable; Use of intra-articular injections or arthroscopy of the index knee within 3 months prior to screening; Evidence of crystalline-induced synovitis in the index knee; History, physical examination, or radiographic results suggestive of acute inflammatory arthritis, rheumatoid arthritis, psoriatic arthritis, septic arthritis, gout, pseudogout, fibromyalgia, systemic lupus erythematosus, or other types of inflammatory arthritis of the index knee; Pain in either knee of neurologic origin;	Signs of clinically important active inflammation in the index knee joint including redness, warmth, and/or a large, bulging effusion with the loss of normal contour at the screening and/or baseline visits; History of acute inflammatory arthritis or pseudogout of the study joint; Cancer/metastatic disease; Systemic or intra-articular corticosteroid use within 60 days of the Screening visit; Known sensitivity to any of the ingredients listed in the treatments under investigation; Significant medical and skin conditions which, in the opinion of the investigator, will interfere with study participation and patch adherence; Replacement of the knee joint.

### Randomization

A total of 500 patients, 100 in each hospital, will be recruited from eligible patients. They will be randomly and blindly assigned to 5 different treating groups through the globally multicentre online randomization facility established by China Academy of Chinese Medical Sciences http://210.76.97.192:8080/cjbyj/.

### Intervention

On Day 0 (Visit 1), patients will begin treatment with either herbal drug, herbal patch, acupuncture, Tuina, or integrative approach. On Day 14, patients will return for an interim visit (Visit 2). On Day 28, the final visit will take place (Visit 3). Thus, the duration of treatment is 4 weeks.

### Oral administration of herbal drug

Participants in this group will receive counter osteophytes capsule (approval NO:Z10980006, manufactured by Jiangsu Kanion Pharmaceutical Co., Ltd) at a daily dose of 5.25 g.

### Topical application of herbal patch

The product, Fufang Zijin patch (approval NO:Z19991106, manufactured by Shanghai LEY's Pharmaceutical Co., Ltd), will be applied at bed time to the right or left knee selected for the study and removed at approximately 8 hours after application (not to exceed 12 hours of exposure time). The location (right or left knee) and time of patch application and removal will be recorded daily in a diary for compliance purposes.

### Tuina

The manipulation will be performed according to established standard operation procedure (SOP) (Table 2).

**Table 2:** Tuina therapy for knee OA

Sequence number	Tuina manipulation
1	The therapist using grasping or rolling applies to the back of the thigh and the crus for 2 minutes.
2	Pushing, kneading or pushing with one-finger meditation on popliteal fossa for 2 minutes.
3	Rolling applies to tensor fascia lata, quadriceps and adductors in the limb for 3 minutes.
4	Rubbing, kneading or pushing with one-finger meditation applies to the Dubi(ST35), Neixiyan(EX-LE5), Ashi points, and each acupoint is about 40 seconds.
5	Gently Pushing the patella to the upward, downward, inward and outward directions for several times, then to the extreme limit of patella and maintaining for 2 and 3 seconds. Repeat 3 times.
6	Pulling the knee. The assistant helps fix the distal thigh, and the therapist uses hands to hold the distal leg and pull for 2 seconds. Repeat 5 times.
7	Passive flexion and extension, adduction and abduction of hip and knee joint to the utmost degree that patient can stand. Repeat 3 times.

Description: The power of tuina manipulation requires evenly and gently application to the degree that patients feel comfort and tolerance. In operations 1 and 2, the patient remains in the prone position. In operations 3 through 7, the patient assumes the supine position.

Dose: about 20 minutes each time and 2 times per week.

### Acupuncture

Acupuncture will be performed according to previously reported SOP [13,14].

### Individually integrated Traditional Chinese Medicine approach

The approach consist of basic treatment of oral counter osteophytes capsule and varying use of Tuina, acupuncture and herbal patch based on the severity of patient's symptoms. The differing individually integrated TCM treatments are shown on the left column of Table 3. The treatment combination (called "approach content" in Table 3) used depends on the WOMAC characteristics as listed in the right column of Table 3. The baseline WOMAC determines the initial treatment, and the ongoing WOMAC later in the trial determines the later ongoing treatments in this arm of the trial.

**Table 3:** Individually Integrated TCM approach for knee OA

Approach content	WOMAC scoring
counter osteophytes capsule	all scoring < 40 mm
counter osteophytes capsule + acupuncture	any one in 5 pain subscales ≥ 40 mm
counter osteophytes capsule + Fufang Zijin patch	any one in 2 stiffness subscales ≥ 40 mm
counter osteophytes capsule + Tuina	any one in 17 physical function subscales ≥ 40 mm
counter osteophytes capsule + acupuncture + Fufnag Zijin patch	both at least one in 5 pain subscales and at least one in 2 stiffness subscales ≥ 40 mm
counter osteophytes capsule + acupuncture + Tuina	both at least one in 5 pain subscales and at least one in 17 physical function subscales ≥ 40 mm
counter osteophytes capsule + Fufang Zijin patch + Tuina	both at least one in 2 stiffness subscales and at least one in 17 physical function subscales ≥ 40 mm
counter osteophytes capsule + acupuncture + Fufang Zijin patch + Tuina	at least one in 5 pain subscales and one in 2 stiffness subscales and one in 17 physical function subscales ≥ 40 mm

Patients in all arms are allowed paracetamol as rescue medication for breakthrough pain at a dose of 500 mg up to a maximum of 2 g per day. The use of rescue medication should not exceed 3 days in each week and should not be taken within 48 hours of a follow up visit. A count of returned tablets will be done at each visit.

### Quality assurance

To ensure that treatments are of a high standard and delivered in accordance with the trial protocol, therapists responsible for application of Tuina and acupuncture will attend a two-day training workshop on the delivery of the treatment programs. They will also be provided with a written protocol and standardized recording documents. In addition, all treatments provided to patients will be carefully recorded. Although patients, investigators, and therapists administering treatment will not be blinded, those assessing the patients for primary and secondary endpoints will be blinded to patient treatment assignment. The investigators, therapists and assessors are different people.

### Endpoints

The primary efficacy endpoint of the study will be Western Ontario and McMaster Universities Index of OA (WOMAC) [15] total score at Day14 and Day 28.

Secondary efficacy endpoints are : i) patient global assessment of response to treatment; ii) patient global assessment of OA condition; iii) WOMAC pain, stiffness, and physical function subscales; iv) investigator global assessment of response to treatment; v) investigator global assessment of OA condition; vi) TCM assessment of OA condition measured by syndromes questionnaire (Table 4); vii)SF-36 assessment.

**Table 4:** Syndromes questionnaire for TCM assessment of knee OA condition

Tongue color (choose one with √ or note for others)					
pale tongue □ pale red tongue □	red tongue □		teeth-marked tongue □	others (please note)	

Tongue fur (choose one with √ or note for others)					

white fur □ yellow fur □	thin fur □		thin fur □	others (please note)	

Pulse diagnosis (choose one with √ or note for others)					

floating pulse □ sunken pulse □ slow pulse □	rapid pulse □ fine pulse □		slippery pulse □ string-like pulse □	others (please note)	

Knee joint situation (mark with √ for yes or no)					

morning stiffness	no □	yes □	cold pain	no □	yes □
pain of unfixed location	no □	yes □	stabbing pain	no □	yes □
joint swelling	no □	yes □	muscle atrophy	no □	yes □

Whole body situation (mark with √ for yes or no)					

easy to sweat at daytime	no □	yes □	easy to sweat at nighttime	no □	yes □
sloppy stool	no □	yes □	hard bound stool	no □	yes □
dry mouth	no □	yes □	profuse dreaming	no □	yes □
reddish yellow urine	no □	yes □	frequent urination	no □	yes □
vexing heat in the extremities	no □	yes □	poor libido	no □	yes □
deafness	no □	yes □	torpid intake	no □	yes □
dizzy vision	no □	yes □	tinnitus	no □	yes □
lack of strength	no □	yes □	somnolence	no □	yes □
lassitude of spirit	no □	yes □	inability to sleep	no □	yes □

### Safety assessments

Spontaneously reported adverse events will be recorded throughout the study. Vital signs will be monitored at every visit. Laboratory investigations including haematology, blood chemistry and urinalysis will be performed at Day 0 and Day 28, and repeated on a need basis. For all adverse events the intensity, relation to test drug, and actions taken will be recorded. All adverse events including reports of skin irritation will be followed until resolution of the event occurs.

### Sample size

Sample size calculations were based on published data [5,14,16]. The mean differences in WOMAC scores before and after treatment with the integrative TCM approach, counter osteophytes capsule, Fufang Zijin patch, acupuncture and Tuina are 11 (SD = 5), 12 (SD = 6), 9.34 (SD = 4.09), 10.17 (SD = 3.85) and 8.89 (SD = 4.32), respectively. Using these data we estimated that 420 patients would be sufficient to give 0.8 power at 0.05 alpha level. Allowing for dropout, we plan to enroll 500 patients.

### Analysis plan

The Biometrics and Clinical Data Systems Department at the China Academy of Chinese Medical Sciences will be responsible for the data management and statistical analysis of this study.

Demographic and baseline variables will be compared across treatment groups using Analysis of Variance (ANOVA) for continuous variables and the chi-square test for categorical variables. If the expected number of subjects within a specific category is sufficiently small, Fisher's exact test will be used in place of the chi-square test.

### Efficacy analysis

The primary efficacy analysis will be based on the full analysis set, defined as all randomized subjects who have baseline and at least one post-baseline efficacy assessment. OA trials are prone to dropouts related to treatment (e.g., inadequate response or intolerance) and because such dropouts will not be at random, thus standard imputation methods are inapplicable. Therefore, we will conduct sensitivity analysis described below wherein missing data are imputed to increasingly adverse degrees to explore whether the primary result still stands. As a supportive analysis, efficacy will also be assessed based on the per protocol set, defined patients in the full analysis set who have no major protocol violation. Statistical comparisons for total and subscale scores based on the WOMAC will be made using a repeated-measure mixed model with terms for treatment, day, center, and baseline value as covariate. The within-subject covariance structure will be assumed unstructured. Treatment-by-day and day-by-baseline interactions will be included in order to compare the treatment difference at each assessment time point as well as for the average across time points. The treatment difference and its 95% confidence interval will be estimated from this model. The same approach will be used for analyzing the Patient Global Assessment of OA condition and the Investigator Global Assessment of OA condition. A similar approach will be used for analysis of Patient and Investigator Global Assessments of response to treatment, but without inclusion of a baseline covariate and associated interaction terms.

### Sensitivity analysis

In this analysis missing data for patients prematurely discontinuing from the trial (or for patients for other reasons not included in the full analysis set) are generated by random draw from the completer patient data, then adjusted as described below. The analysis is described for a situation where treatment A is statistically significantly superior to treatment B by the primary efficacy analysis. The treatment effect is the difference of the WOMAC change in the treatment A and the WOMAC change in treatment B. Call this treatment effect difference D. Four scenarios are investigated, corresponding to the loss of 25%, 50%, 75%, and 100% of the treatment effect difference. Patients that prematurely discontinued from treatment A will have their WOMAC change values determined by random draw (with replacement) from the treatment A completers, then these values are adjusted downward by 1/8*D, 2/8*D, 3/8*D, then 4/8*D, for the 1^st^, 2^nd^, 3^rd^, and 4^th ^scenario, respectively. Treatment B dropouts will have their random draw values adjusted upward sequentially by the same factors (1/8*D, 2/8*D, 3/8*D, then 4/8*D). The four scenarios, therefore, are:

#1-Assuming loss of 25% of the treatment effect; this is the least adverse scenario.

#2-Assuming loss of 50% of the treatment effect seen in completers.

#3-Assuming loss of 75% of the treatment effect seen in completers.

#4-Assuming loss of 100% of the treatment effect; this is the most adverse scenario.

Because the analysis involves stochastic variables, it will be repeated 100 times in order to better estimate the means and approximate 95% confidence intervals.

The following treatment comparisons will be performed based on the repeated measures mixed model and sensitivity analysis:i) Comparison at each of Days 0, 14, and 28;ii) Comparison of the Day 14 and 28 averages, using the time-averaged Day 14 and 28 scores estimated within the model framework;iii) Comparison of the average over all post-treatment days, using the time-averaged Day 14 and 28 scores estimated within the model framework;iv)All statistical tests will be two-sided. The primary endpoint and key secondary endpoints will be tested.

### Data integrity and management

Data will be stored electronically on a database with secured and restricted access. Data transfer will be encrypted and any information capable of identifying individuals removed.

### Withdrawal

A participant will be considered to have withdrawn from the trial when consent is revoked (except to ask permission for a determination of a final withdrawal visit WOMAC score) or if the participant cannot be contacted or located. If this occurs, no further assessments will be performed. Participants will not be withdrawn from the trial for protocol violations. Withdrawal due to adverse event should be distinguished from withdrawal due to insufficient response. When a subject withdraws due to a serious adverse event, the serious adverse event must be reported in accordance with the reporting requirements.

### Monitoring

The trial will be overseen and monitored by a program manager. The program manager will visit each site to examine trial procedures, ensure data quality and monitor compliance with the trial protocol.

## Discussion

The biggest limitation of this study is the lack of placebo control. It should be noted that the aim of this trial is not to estimate the efficacy of treatments used, but to demonstrate that individually integrated TCM approach is superior to individual component alone. This aim arises from clinical experience. The components of these treatments have been used for centuries. TCM has over 2000 years' application in China, usually as "one method applied in a whole course of treatment". However, even though the individual approach, based on different features of OA, is recommended, there is no evidence that it is superior to a treatment component used alone.

In an OA trial, a statistically significant result might be due to the dropout pattern rather than a true treatment effect, so we are attempting to collect data at the time of discontinuation to enable better comparisons of those completing versus those discontinuing. Additionally, we are conducting a formal sensitivity analysis using imputed data for discontinued patients to determine whether a positive result remains positive in scenarios where part or all of the treatment effect difference seen in the completers is lost in the dropout patients. To our knowledge, this is the first trial in a chronic symptomatic disorder to explicitly specify this type of sensitivity analysis to address trial dropouts.

Our results will be conditional on the population recruited, and the patients we recruited, requiring pain intensity of 20 mm or more on visual analogue scales (VAS), may have larger or smaller responses to treatment compared to those without such a restriction. For example, it was recently shown that use of a "flare" to define trial eligibility in OA alters, on average, response to treatment [17]. Another limitation is no double-blinding. Although it is difficult to carry out double-blinding in this design, we blind those clinicians conducting the assessments to decrease possible bias. The study also lacks long-term follow-up and evaluation. However, the duration chosen in the present study is consistent with acute OA flare episodes; and the study is long enough to document a rapid onset of a treatment modality and its efficacy sustained for 4 weeks. The protocol has a provision for rescue medication although its use is discouraged.

OA diagnosis is made based on objective radiological criteria along with excluding inection or gout, and it made using the medical history and physical exam. The WOMAC index is a validated scale that uses 24 parameters to assess OA of the hip or knee with focus on Pain, Stiffness, Physical Function, Social Function and Emotional Function [18,19]. The design of this study is consistent with both global clinical research standards and use of TCM criteria for evaluating efficacy and safety. One important principle guiding TCM therapy is to use drug treatment according to "Syndromes". Syndrome is different from the disease name, since it is a summarized analysis of the symptom data from patients' whole situation using TCM methods. It may not only include local symptoms which are part of the disease but also symptoms from the whole body situation. Given the differences in the whole body situation in different people, it is natural that OA, a kind of disease, has different Syndromes. In TCM, OA is known as Bi, Wei, Ganshen kuixu, or Jinmai yuzhi Syndrome and so on [20].

WOMAC questionnaires are detailed in the description of local symptoms of OA, while the Syndromes questionnaires more emphasizes the body's whole situation. Thus, the result of this research could also provide information on the correlation of symptoms-analysis gathered from WOMAC and Syndromes of TCM in different phases (mild, moderate or severe) of OA which may be helpful for predicting severity of OA using TCM methods.

Given the use of two manipulative methods (acupuncture, Tuina) in this study, patients' compliance and response to treatment is critical to the treatment effect. WOMAC subscale analysis will provide information on which OA symptom (pain, stiffness, or function) was more responsive to which treatment. Since this is an East meet West study, outcome measures capture both OA symptoms and the whole body TCM assessment according to TCM theory. We also include a standard quality of life measure, the SF-36, as in many other OA trials [21,22].

## List of abbreviations

(OA): Osteoarthritis; (RCT): randomized controlled trials; (TCM): Traditional Chinese Medicine; (WOMAC): Western Ontario McMaster Universities Osteoarthritis Index; (SF-36): short-form 36; (ACR): American College of Rheumatology; (SOP): Standard Operation Procedure; (VAS): visual analogue scales.

## Competing interests

The authors declare that they have no competing interests.

## Authors' contributions

ZHS and CYL conceived of the study, and participated in its design and coordination. CYL, LFY, XSJ, GJH, XZW, LG, PJ, GCQ and LT performed research. CYL and SYY wrote and revised the manuscript. All authors gave final approval of the version to be submitted.

## References

[B1] Dougados M Osteoarthritis: more evidence for non-pharmacological OA therapy Nat Rev Rheumatol 2009 5 597 598 10.1038/nrrheum.2009.214 19865089

[B2] Felson DT Developments in the clinical understanding of osteoarthritis Arthritis Res Ther 2009 11 203 10.1186/ar2531 19232065 PMC2688214

[B3] Long L Soeken K Ernst E Herbal medicines for the treatment of osteoarthritis: a systematic review Rheumatology (Oxford) 2001 40 779 793 10.1093/rheumatology/40.7.779 11477283

[B4] BL L Clinical report of counter-osteophytes capsule on the treatment of OA in 1,000 cases Liaoning Traditional Chinese Med J 1982 3 40 41

[B5] Cao Y Shi Y Zheng Y Shi M Lo SK Blood-nourishing and hard-softening capsule costs less in the management of osteoarthritic knee pain: a randomized controlled trial Evid Based Complement Alternat Med 2005 2 363 368 10.1093/ecam/neh104 16136214 PMC1193545

[B6] Ramsey SD Spencer AC Topolski TD Belza B Patrick DL Use of alternative therapies by older adults with osteoarthritis Arthritis Rheum 2001 45 222 227 10.1002/1529-0131(200106)45:3<222::AID-ART252>3.0.CO;2-N 11409661

[B7] Perlman AI Sabina A Williams AL Njike VY Katz DL Massage therapy for osteoarthritis of the knee: a randomized controlled trial Arch Intern Med 2006 166 2533 2538 10.1001/archinte.166.22.2533 17159021

[B8] Zhang W Moskowitz RW Nuki G Abramson S Altman RD Arden N Bierma-Zeinstra S Brandt KD Croft P Doherty M OARSI recommendations for the management of hip and knee osteoarthritis, part I: critical appraisal of existing treatment guidelines and systematic review of current research evidence Osteoarthritis Cartilage 2007 15 981 1000 10.1016/j.joca.2007.06.014 17719803

[B9] Ezzo J Hadhazy V Birch S Lao L Kaplan G Hochberg M Berman B Acupuncture for osteoarthritis of the knee: a systematic review Arthritis Rheum 2001 44 819 825 10.1002/1529-0131(200104)44:4<819::AID-ANR138>3.0.CO;2-P 11315921

[B10] Kwon YD Pittler MH Ernst E Acupuncture for peripheral joint osteoarthritis: a systematic review and meta-analysis Rheumatology (Oxford) 2006 45 1331 1337 10.1093/rheumatology/kel207 16936326

[B11] Zev Shainhouse J OARSI guidelines for hip and knee OA: deciphering the topical drug melange Osteoarthritis Cartilage 2008 16 1586 1587 author reply 1588 10.1016/j.joca.2008.04.016 18524636

[B12] Belo JN Berger MY Koes BW Bierma-Zeinstra SM The prognostic value of the clinical ACR classification criteria of knee osteoarthritis for persisting knee complaints and increase of disability in general practice Osteoarthritis Cartilage 2009 17 1288 1292 10.1016/j.joca.2009.04.002 19410034

[B13] Lao L Berman B [Evaluating the effects of acupuncture on knee osteoarthritis: a stepwise approach to research, University of Maryland experience] Zhong Xi Yi Jie He Xue Bao 2005 3 421 425 10.3736/jcim20050601 16282047

[B14] Berman BM Singh BB Lao L Langenberg P Li H Hadhazy V Bareta J Hochberg M A randomized trial of acupuncture as an adjunctive therapy in osteoarthritis of the knee Rheumatology (Oxford) 1999 38 346 354 10.1093/rheumatology/38.4.346 10378713

[B15] Faik A Benbouazza K Amine B Maaroufi H Bahiri R Lazrak N Aboukal R Hajjaj-Hassouni N Translation and validation of Moroccan Western Ontario and McMaster Universities (WOMAC) osteoarthritis index in knee osteoarthritis Rheumatol Int 2008 28 677 683 10.1007/s00296-007-0498-z 18092169

[B16] Bhattacharyya T Gale D Dewire P Totterman S Gale ME McLaughlin S Einhorn TA Felson DT The clinical importance of meniscal tears demonstrated by magnetic resonance imaging in osteoarthritis of the knee J Bone Joint Surg Am 2003 85-A 4 9 12533565 10.2106/00004623-200301000-00002

[B17] Trijau S Avouac J Escalas C Gossec L Dougados M Influence of flare design on symptomatic efficacy of non-steroidal anti-inflammatory drugs in osteoarthritis: a meta-analysis of randomized placebo-controlled trials Osteoarthritis Cartilage 2010 18 1012 1018 10.1016/j.joca.2010.04.005 20417293

[B18] Bellamy N Buchanan WW Goldsmith CH Campbell J Stitt LW Validation study of WOMAC: a health status instrument for measuring clinically important patient relevant outcomes to antirheumatic drug therapy in patients with osteoarthritis of the hip or knee J Rheumatol 1988 15 1833 1840 3068365

[B19] Ackerman I Western Ontario and McMaster Universities Osteoarthritis Index (WOMAC) Aust J Physiother 2009 55 213 10.1016/S0004-9514(09)70088-1 19736686

[B20] Shen ZY [Basic theory of traditional Chinese medicine] Zhongguo Zhong Xi Yi Jie He Za Zhi 1997 17 643 644 10322840

[B21] Jones JG Leighton F Comparison of WOMAC with SF-36 for OA of the knee or hip Ann Rheum Dis 2002 61 182 183 10.1136/ard.61.2.182 11796410 PMC1754004

[B22] Odland AN Callaghan JJ Liu SS Wells CW Wear and lysis is the problem in modular TKA in the young OA patient at 10 years Clin Orthop Relat Res 2011 469 41 47 10.1007/s11999-010-1429-y 20568028 PMC3008910

